# Developing IBD Outcome Effect Size Thresholds to Inform Research, Guidelines, and Clinical Decisions

**DOI:** 10.1093/ibd/izaf085

**Published:** 2025-05-13

**Authors:** Morris Gordon, Nader Shaban, Vasiliki Sinopoulou, Sudheer Vuyyuru, Shellie Radford, Fernando Magro, Alessandro Armuzzi, Laurent Peyrin-Biroulet, Vipul Jairath, Gordon Moran

**Affiliations:** Biomedical Evidence Synthesis and Translation, University of Central Lancashire, Lancashire, Preston, United Kingdom; London Health Sciences Centre, London, Ontario, Canada; Biomedical Evidence Synthesis and Translation, University of Central Lancashire, Lancashire, Preston, United Kingdom; London Health Sciences Centre, London, Ontario, Canada; Department of Gastroenterology, University of Western Ontario, London, Ontario, Canada; Nottingham NIHR Biomedical Research Centre, University of Nottingham, Nottingham, Nottinghamshire, United Kingdom; CINTESIS@RISE, Faculty of Medicine, University of Porto, Porto, Portugal; IBD Center, IRCCS Humanitas Research Hospital, Rozzano, Milan, Italy; Department of Biomedical Sciences, Humanitas University, Pieve Emanuele, Milan, Italy; INFINY Institute, INSERM NGERE, Department of Gastroenterology, CHRU de Nancy, Université de Lorraine, Vandœuvre-lès-Nancy, France; London Health Sciences Centre, London, Ontario, Canada; Department of Gastroenterology, University of Western Ontario, London, Ontario, Canada; Nottingham NIHR Biomedical Research Centre, University of Nottingham, Nottingham, Nottinghamshire, United Kingdom

**Keywords:** IBD, Inflammatory Bowel Disease, clinical remission, endoscopic remission, Ulcerative colitis, Crohn’s disease

## Abstract

**Background:**

When designing clinical trials, interpreting trial outcomes for guideline development or sharing decisions with patients in clinical practice, the clinical outcomes used and the implicit choices on what constitutes a clinically significant finding can vary greatly. This can lead to diversity or even inequity in care offered to patients with inflammatory bowel disease (IBD). The GRADE approach to guideline development has proposed a process to address this prospectively to solve these issues, but this has never been used in IBD. We aimed to develop the first international consensus set of outcome thresholds to establish their use in Crohn’s disease and ulcerative colitis.

**Methods:**

A Delphi methodology was used to develop a consensus. An online survey was conducted by inviting stakeholders from the British Society of Gastroenterology through a 2-phase process. Participants were asked to select important clinically relevant outcomes and were asked about what magnitude of the effect that they consider large, moderate, small, or trivial for each clinical trial outcome in line with the GRADE guidance. The results were fed back to all participants to ensure consensus agreement. Then, further surveys were sent to Europe and North America to ensure validity and international triangulation of the dataset. Data are presented as mean ± SD.

**Results:**

A total of 131 clinical stakeholders participated, including clinicians, IBD nurses, and a small number of patients with IBD. Clinical remission and serious adverse events were considered the most critical outcomes for Crohn’s disease, while clinical remission and endoscopic remission were considered the most critical outcomes for ulcerative colitis. The consensus results for thresholds of small, moderate, and large outcome effect sizes were agreed on as follows: clinical remission, 11 ± 6%, 20 ± 8%, and 31 ± 13%; endoscopic remission, 9 ± 5%, 17 ± 9%, and 28 ± 14%; and serious adverse events 6 ± 6%, 11 ± 9%, and 17 ± 12%, respectively. No significant differences were observed for responses for each condition.

**Conclusions:**

This is the first study to develop a consensus on magnitude thresholds for outcomes in IBD. These thresholds have been used in the development of the 2024 British Society of Gastroenterology guidelines for the management of IBD but can and should also be used by study designers and, most importantly, by clinicians when discussing evidence with patients as part of shared decision making. Future work to validate these findings globally and with other groups, including patients, is needed.

**Key messages**

- What is already known?

• Existing clinical guidelines in inflammatory bowel disease acknowledge that certain outcomes are of greater importance for clinical practice, and within each outcome there is a minimum clinically important difference, below which findings are of little significance.

- What is new here?

• This study has developed the first international consensus prioritization of outcomes and agreement on detailed thresholds for magnitude of outcomes at the levels of trivial, small, moderate, and large for each outcome.

- How can this study help patient care?

• This agreement can aid shared decision making and transparent clinical guideline development, ensuring that practitioners can consistently communicate to patients the expected impact of given therapies on key outcomes.

## Introduction

In 2021, the British Society of Gastroenterology (BSG) formed a guideline development group (GDG) led by multidisciplinary clinical experts within the United Kingdom alongside expert GRADE (Grading of Recommendations Assessment, Development, and Evaluation) methodologists from the Cochrane Gut group to produce the 2024 inflammatory bowel disease (IBD) guidelines.^1^ Core to these approaches and informing all elements of decision making, especially in GRADE, is an agreement on the range and relative importance of outcomes to be used and the specific measures for each outcome that have the most consensus.^2^

There are several outcomes of interest in IBD. In 2021, the International Organization for the Study of Inflammatory Bowel Disease undertook evidence synthesis and a Delphi consensus exercise to define the important outcomes in Crohn’s disease and ulcerative colitis.^3^ They ranked the importance of short-term treatment goals in Crohn’s disease: clinical remission, endoscopic response, clinical response, and normalization of biochemical markers. In ulcerative colitis, this hierarchy was led by clinical remission and response, endoscopic response, and normalization of biochemical markers. Clarification of these treatment targets with expected timelines to achieve them is of importance to the clinical and academic community, as it allows clinicians to assess treatment response appropriately, helps academic communities and industry partners to design relevant clinical trials, and helps people living with IBD to set their expectations when receiving medical treatment.

Another core approach in GRADE decision making is to develop explicit thresholds for interpretation of effect sizes of outcomes to inform in sample size estimations. The clinically relevant differences in critical and important outcomes when comparing IBD treatments are unclear. The importance of a threshold is becoming recognized in guidelines for IBD, but this is commonly set as a single dichotomous cutoff with no rationale informing its use, if considered at all.^4,5^ The validity of the cutoff as well as the limitations of such a dichotomous concept of “significance” are significant concerns with such an approach. Moreover, a quarter of randomized controlled trials on IBD interventions have no power calculation, and a further quarter (often including more recent trials) base these on arbitrary minimally clinical important differences (MCIDs), which are essentially a minimum “threshold” but have no objective or evidence-based justification.^6^ When an MCID is reported, the smallest reported is 10% but overall, these range enormously across IBD studies and are only ever reported based on a single outcome. The differences reported in power calculations rarely match the actual differences achieved by wider studies in practice, making a lot of these studies at risk of being underpowered or imprecise for certain outcomes.^7^

It is key for all clinical decision makers working with evidence, and especially those deciding on therapies with patients, to consider 2 questions: (1) “How substantial are the desirable anticipated effects (health benefits)?” and (2) “How substantial are the undesirable anticipated effects (health harms)?”^7^ Answering these questions needs a similar understanding of what magnitude of health benefits or health harms to inform decisions. Despite the popular use of thresholding to achieve this in healthcare research, with implementation for guideline decision making by GRADE,^8-12^ wider establishment across healthcare is lacking. Recently, studies have confirmed the utility and validity of this approach,^7^ but to date it has not been employed in gastroenterology or in the context of a large and complex guideline process. When designing the new UK IBD guidelines, patient representatives highlighted how vital they believed this clarification is for clear decision making in a shared fashion.^1^

What is urgently needed is a widespread agreement of the critical and important outcomes in Crohn’s disease and ulcerative colitis, including efficacy as well as safety outcomes, and more importantly a definition for each outcome regarding what trivial, small, medium and large thresholds entail. We set out use a Delphi approach from both the United Kingdom and a wider international audience to produce the first expert consensus agreement on outcome measures and thresholds of effect size in IBD.

## Methods

An online Delphi process was undertaken.^13^ This was delivered online through using Survey Monkey (www.surveymonkey.com) with 2 phases. The first phase in the United Kingdom included 3 rounds to reach a Delphi consensus. The second round was international and was a single Delphi round to triangulate the findings of the UK phase.

The first phase of delivery was through was within the United Kingdom to the BSG GDG. After an initial test was conducted with the core members of the GDG, the survey was opened to the wider GDG for round 1, made up of secondary and tertiary gastroenterologists, pediatricians with an interest in IBD, IBD nurses, allied health professionals with an interest in IBD, and IBD patient and user representatives. These GDG members responded to an open call from BSG to members across all their professional groups and Crohn’s and Colitis UK, the largest UK charity for IBD, to their list of patients and stakeholders with an interest in research activities. No specific training on IBD was offered to participants. Participants were asked to provide demographic and professional information. They were then asked to complete 3 main sections within the survey with an estimated completion time of 25 minutes. It was made clear that participants should only answer questions that were relevant to their expertise. The results from the first round were collated and findings sent out in a round 2 to confirm agreement. A virtual face-to-face discussion was held in May 2023 with the GDG to achieve agreement on the final consensus items and thresholds. The international element of the study was performed next to ensure triangulation and convergence of findings, with further rounds only planned if disagreement was found. The outcomes of the first two rounds were not shared with international participants prospectively.

Following adoption by the GDG, the survey was advertised to the international IBD community in North America and Europe to obtain a wider consensus on the effect thresholds. This was undertaken by approaching key opinion leaders in Italy (A.A.), Portugal (F.M.), France (L.P.-B.), and Canada (V.J.) and supporting these colleagues in distributing the survey through their national specialty contacts.

### Defining the Critical and Important Outcomes in Crohn’s Disease and Ulcerative Colitis

In this section, the participants were asked to determine which of 13 outcomes commonly observed in clinical trials and practice are deemed critical or important to the IBD community. These included clinical remission, clinical response, endoscopic remission, endoscopic response, histological remission, histological response, biochemical remission, biochemical response, radiological remission, radiological response, total adverse events, serious adverse events, and withdrawal due to adverse events. They were tasked with choosing up to 7 outcomes (based on Cochrane and GRADE guidance)^14,15^ that they felt were either critical or important. Participants were asked to complete this section for Crohn’s disease and ulcerative colitis. Seven outcomes in total were chosen as either “critical” or “important,” and the rest were to be chosen as “do not consider.” Further clarifying definitions of these common terms were not used, as these vary within the field. The purpose of this section was to obtain an internally consistent consensus on which outcomes should carry the most weight when presented with data.

Moreover, during this exercise participants were asked to decide on their preferred definition of that outcome. The options provided were as finalized in out BSG IBD guideline methodology published elsewhere.^1^ For completeness, for radiological and histological remission, and reflecting the nonexpert nature of the audience, participants were only asked to vote on the preferred scoring system, with no exact categorical definitions provided. Namely, for radiological outcomes: MARIA (Magnetic Resonance Index of Activity) score, shortened MARIA score, and the Crohn’s disease magnetic resonance imaging index. As for histological outcomes these were the Geboes score, the Nancy histological index, and the Robarts histological index.

### Defining Explicit Thresholds

In the final section of the survey, the participants were asked to decide at what thresholds are the effects of interventions deemed trivial, small, moderate, or large when compared with other interventions or placebo, with trivial implying no difference to the comparator. The participants were asked to decide on the magnitude of thresholds for the outcomes that they previously encountered in section 1 of the survey. All outcomes were dichotomous, and so the choices made were in percentage absolute terms when compared with placebo or control therapy. They were shown the example of a 10% threshold for small in clinical remission. When compared with a placebo, a therapy would need to lead to an addition 10 people out of 100 reaching clinical remission for the effect size to be considered small. Any therapy leading to less would be considered trivial and so no different to placebo. For each outcome and for each of the categories for thresholds, they had to propose their own threshold level. It was clarified that the thresholds did not need to be linear, with the only limits that all thresholds exist between 0% and 100%. At this stage in the survey, respondents were asked not to consider treatment timelines when deciding on thresholds.

### Data Analysis

Findings are presented numerically with percentage data provided wherever possible. Where relevant, data are presented as mean ± SD. Due to the descriptive nature of the project, no a priori hypothesis was set out and no attempt at statistical comparison was undertaken. The study protocol was reviewed and approved by the [HEALTH] Ethics Review Panel at the University of Central Lancashire (0417). Participants were able to proceed with the survey after providing informed consent.

## Results

Between spring 2023 and summer 2024, a total of 131 Delphi responses were received. The results from the first round were of high convergent agreement, and so the collated outcomes were sent to the GDG respondent in round 2 for agreement including a further 89 responses. Thereafter, the questionnaire was sent to international colleagues, collecting 42 further responses (21 from Canada and 21 from Europe). Respondent roles were provided in 128 of 131 responses. These were clinicians with an interest in IBD in the majority (114 of 128), with 4 pharmacists, 4 IBD nurses, and 6 people living with IBD providing responses. Clinicians with an interest in IBD were defined as adult gastroenterologists practicing in a secondary or tertiary care setting who review IBD patients on a weekly basis.

### Critical and Important Outcomes

**Figure 1.**
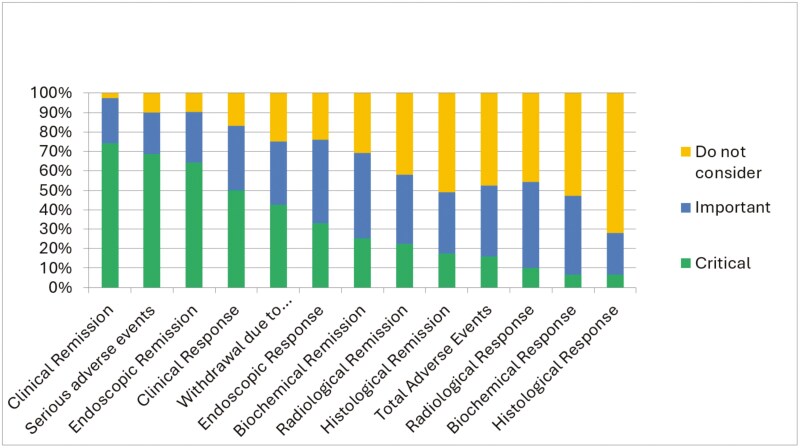
Critical and important outcomes in Crohn’s disease.

**Figure 2.**
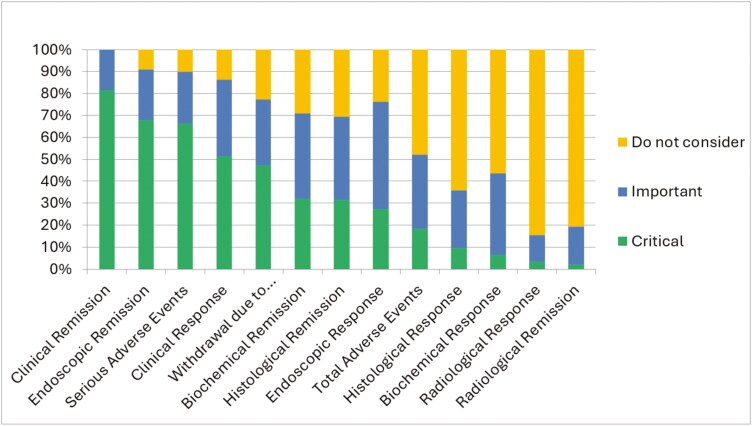
Critical and important outcomes in ulcerative colitis.

The most critical outcomes in Crohn’s disease were clinical remission (74.4% [n = 87 of 117]) and serious adverse events (68.8% [n = 77 of 112]), while radiological and histological remission and response, biochemical response, and total adverse events not considered as important (Figure 1). The most critical outcomes in ulcerative colitis were clinical remission (81.4% [n = 92 of 113]) and endoscopic remission (67.9% [n = 76 of 112]), while radiological outcomes; endoscopic, histological, and biochemical response; and total adverse events were not deemed important (Figure 2). Full numeric data including percentage values, absolute values, and number of responses per question can be found in Supplementary Tables 1 and 2.

### Outcome Definitions

For defining remission in Crohn’s disease, the majority (49.0% [n = 49 of 100]) of respondents preferred a Harvey-Bradshaw index <5, with (88.5% [n = 85 of 96]) respondents favoring a fecal calprotectin of <250 µg/g to define remission. Similarly, the majority (73.2% [n = 41 of 56]) of respondents favored a Simple Endoscopic Score for Crohn’s Disease <3 to define remission (Supplementary Tables 3-5).

As for ulcerative colitis, the majority (42.2% [n = 38 of 90]) of respondents preferred a Simple Clinical Colitis Activity Index of 2 or less to define remission, with 94.7% (n = 90 of 95) preferring a fecal calprotectin of <250 µg/g to refine remission. The majority (60.9% [n = 39 of 64]) preferred an Ulcerative Colitis Endoscopic Index of Severity of <2 to define endoscopic remission (Supplementary Tables 6-8).

### Outcomes Thresholds

**Figure 3.**
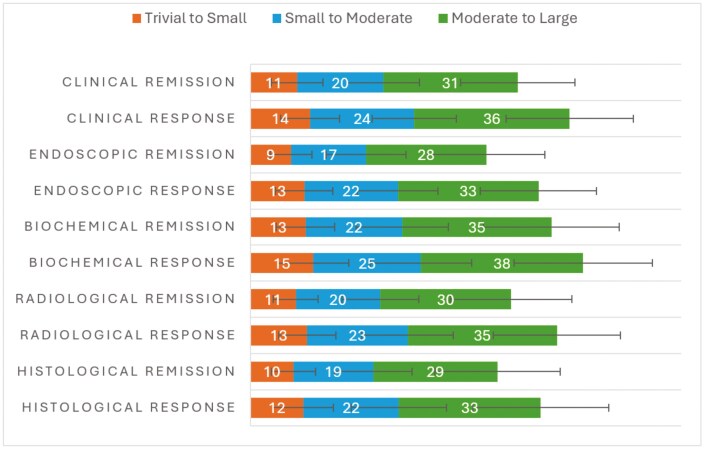
Explicit thresholds for efficacy outcomes in IBD. Data are presented as mean percentages ± SD.

**Figure 4.**
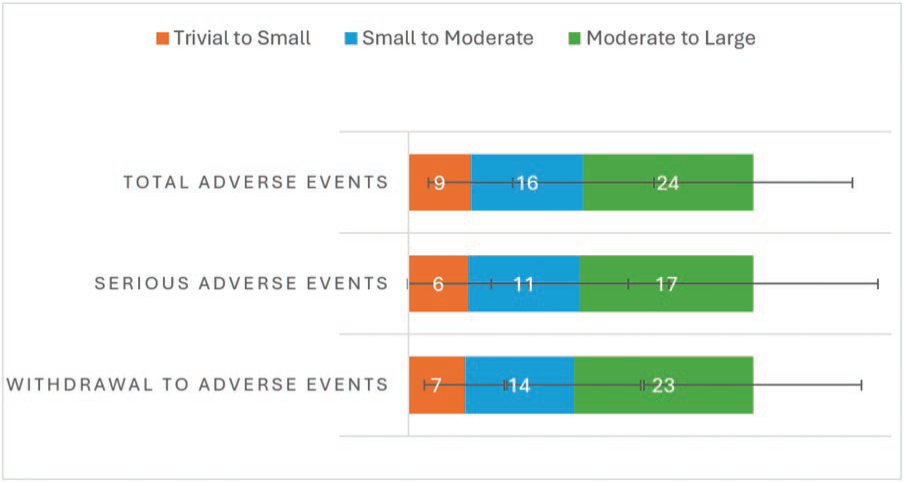
Explicit thresholds for safety outcomes in IBD. Data presented as mean percentages ± SD.

As for the critical outcomes in IBD, trivial to small, small to moderate, and moderate to large thresholds for clinical remission are 11 ± 6%, 20 ± 8%, and 31 ± 13% (n = 98), respectively. For endoscopic remission, trivial to small, small to moderate, and moderate to large thresholds are 9 ± 5%, 17 ± 9%, and 28 ± 14% (n = 87), respectively. For serious adverse events, trivial to small, small to moderate, and moderate to large thresholds are 6 ± 6%, 11 ± 9%, and 17 ± 12% (n = 90), respectively. Figures 3 and 4 illustrate the results for all the efficacy and safety outcomes. Supplementary Table 9 provides numeric data and number of responses for each of the outcomes from overall respondents across regions. Supplementary Tables 10, 11, and 12 show data respective to the geographical regions of the United Kingdom (BSG), Europe, and North America, respectively, with the trends being similar across the regions with no apparent areas of discordance.

## Discussion

This study has established an international stakeholder consensus on priority outcomes for consideration in IBD therapeutic research as well as thresholds for outcome effects. The first UK phase of the study was able to reach consensus within 2 Delphi rounds, which demonstrates the consistency of the judgments made and the face validity of these terms. The international responses were performed independently, and agreement was seen, further enhancing the validity of this first attempt at defining consensus thresholds. For both clinical and endoscopic efficacy outcomes, the initial threshold of “small” was around 10%. This is both consistent with existing literature, as the respondents are proposing that any absolute difference between standard and intervention therapies of <10% is essentially not significant for clinical practice. However, thresholds for moderate and large were much higher. This is different from the existing literature because it is completely novel, as no existing study has set out to follow this GRADE approach and form a range of thresholds. As may be expected, the thresholds were slightly lower for endoscopic outcomes, given that these are perhaps more difficult to achieve, and conversely, thresholds were higher for clinical response than clinical remission, given that this is essentially seen as easier to achieve (large thresholds of 31% for clinical remission, 36% for clinical response, and 28% for endoscopic remission).

Thresholds for efficacy were different to safety outcomes, as they are expected to be. These safety outcomes were much smaller in magnitude and with a tighter spread from small to large, which is a function of their relative significance to stakeholders and especially patients.^16^ There is also a considerable overlap in the defined thresholds for total adverse events, serious adverse events, and withdrawal due to adverse events. It is, at present, not possible to classify this any further, and a larger sample size of both clinicians and people living with IBD is needed to further validate and scrutinize these findings. This is particularly novel, as we are not aware of any other publication that has considered thresholds for significance of safety outcomes in IBD research for stakeholders, and especially for decision making for patients.

The impact of these findings is potentially substantial in a number of contexts. They can support evidence synthesis and systematic review in making imprecision judgments consistently. This is a key element to appraisal evidence in meta-analysis, and without such objectively agreed standards it is absolutely unavoidable that the same review topics will produce different findings. These thresholds can conform to GRADE judgments of evidence and decision making within Evidence to Decision frameworks. Imprecision is a key element of GRADE assessment, and older methods^15^ fail to offer sufficient clinical relevance to their decisions, which this approach resolves. The findings can also support future researchers in powering studies, by considering their chosen outcomes and what magnitude they would want to power for each and in turn produce an overall MCID for power calculation.^6,7^ There is another purpose for researchers, which is the retrospective interpretation of trial results—discussing not just the significance of results, but also their magnitude in the context of these findings.

A final and perhaps most intriguing use of these findings is to inform clinician and patient interpretation of evidence at the individual decision-making level. When discussing treatments with patients, often a dichotomous decision of “whether it works” is discussed, which is analogous to significance levels or *P* values.^17^ GRADE synthesis supports the inclusion of consideration of “how sure we are” on this result, regardless of its significance, but based on certainty/quality.^18^ The addition of information informed by this exercise informs discussion not only of those outcomes of most critical importance to all stakeholders, but also of the expected effect size. This has the potential to change the process of shared decision making, as fundamentally empirical data supporting judgments on health benefits and harms for dichotomous outcomes are not yet available for the Evidence to Decision frameworks.^19^

There are several limitations to the study. The decisions have been made by a significant sample with a wide range of expertise and international contribution. However, increased sample numbers, increased variety of stakeholders, and increased geographical spread of participants (particularly within the global south) will further enhance generalizability of the findings. A greater sample size would allow for a subanalysis of findings based on geographical regions, which may in turn help inform regional stakeholders such as the Food and Drug Administration and European Medicines Agency. It is also key to consider patient and user involvement, and while there was patient representative on the GDG panel who also contributed to this study, this was a low proportion of respondents, and it is unclear whether a wider level of involvement would impact the findings. Previous works focused on patients in the context of discrete choice experiments^20^ have found a broad alignment with the findings of this study in terms of prioritization. However, as the majority of responses were from IBD clinicians, any future work should encompass a much larger patient voice. It is also conceivable that there is no single generalizable set of thresholds internationally, and so without further international consideration of this approach, this remains a risk in interpretation. It is also important to note that the phrasing of the questions is very much focused on outcomes of interest from existing research. As such, using this approach retrospectively is helpful, but it cannot be considered an approach to inform choices of outcomes prospectively, as participants would not consider novel, emerging, or even yet not considered outcomes that may become significant in the future. Similarly, we did not investigate important outcomes such as disability and quality of life, and these are potentially relevant, as they are patient reported. We have homogenized the threshold outcomes for ulcerative colitis and Crohn’s disease. Moreover, these thresholds may differ, taking into consideration the refractory nature of disease activity, disease burden, and most importantly, the timing of when an outcome is attained. The final limitation is related to the potential bias that our worked example of thresholds may have introduced. This was a challenge, as without an example, conceptualization was felt to be difficult. The choice of 10% as a minimum threshold was made, as this is already widely used in practice as a single dichotomous threshold, but readers must consider the impact that these examples may have had as a source of bias.

In summary, we have undertaken an international Delphi exercise in which we observed that clinical remission was the most critical outcome for both Crohn’s disease and ulcerative colitis in an era in which objective outcomes are the fore of IBD research. This observation is similar to the one described a few years ago by the STRIDE (Selecting Therapeutic Targets in Inflammatory Bowel Disease) consortium and highlights the importance of a symptom-free state for both clinicians and people living with IBD. Moreover, for the first time ever, we have defined the trivial, small, medium, and large thresholds for all critical and important outcomes in IBD.

Future research is needed to develop this novel work further. It is suggested that a similar project with much larger sample size and diversity of respondents is undertaken. This could be several national studies or one large international study. The first option does have the advantage of considering if potential local contextual factors modify effect sizes. It is also important to consider the views of patients (a separate exercise with a tailored approach to seek the wider views on these issues from appropriate users is key) and if these converge or diverge from other stakeholders. The advantage of a large global effort would be to consider not local factors, but instead patient factors to modify the thresholds. For example, this could consider the type of IBD, stage of disease and whether patients are bio-naïve or bio-exposed. These would increase the fidelity and utility of the tool. Finally, the use of this resource by researchers to inform sample size calculations is of particular interest, and research as to the utility and validity of the findings for this purpose is needed. This study has been able to produce a consensus set of outcomes and effect size thresholds for IBD. This resource can support evidence synthesis in a consistent fashion, support decision making in guidelines, support shared decision making with patients, and inform sample size calculations for research. Future studies are needed to ensure the validity and generalizability of this set of judgments.

## Supplementary Material

izaf085_Supplementary_Tables

## Data sharing

All of our data, except participant identifiers, are available on reasonable request.
