# Supplementary material for: Developing IBD Outcome Effect Size Thresholds to Inform Research, Guidelines, and Clinical Decisions
Source: Inflamm Bowel Dis. 2025 May 13;31(10):2798–804. doi: 10.1093/ibd/izaf085 (PMC12558582; doi:10.1093/ibd/izaf085)
Supplement: izaf085_Supplementary_Tables [file izaf085_supplementary_tables.docx]

| Supplementary Table 1: Critical and important outcomes for Crohn’s disease raw data  Number of participants who answered = 118 | | | | | | | |
| --- | --- | --- | --- | --- | --- | --- | --- |
|  | Critical | | Important | | Do not consider | | Total |
| Clinical Remission | 74.36% | 87 | 23.08% | 27 | 2.56% | 3 | 117 |
| Serious adverse events | 68.75% | 77 | 21.43% | 24 | 9.82% | 11 | 112 |
| Endoscopic Remission | 64.35% | 74 | 26.09% | 30 | 9.57% | 11 | 115 |
| Clinical Response | 50.00% | 57 | 33.33% | 38 | 16.67% | 19 | 114 |
| Withdrawal due to Adverse Events | 42.57% | 43 | 32.67% | 33 | 24.75% | 25 | 101 |
| Endoscopic Response | 33.03% | 36 | 43.12% | 47 | 23.85% | 26 | 109 |
| Biochemical Remission | 25.23% | 27 | 43.93% | 47 | 30.84% | 33 | 107 |
| Radiological Remission | 22.43% | 24 | 35.51% | 38 | 42.06% | 45 | 107 |
| Histological Remission | 17.31% | 18 | 31.73% | 33 | 50.96% | 53 | 104 |
| Total Adverse Events | 15.84% | 16 | 36.63% | 37 | 47.52% | 48 | 101 |
| Radiological Response | 9.90% | 10 | 44.55% | 45 | 45.54% | 46 | 101 |
| Biochemical Response | 6.73% | 7 | 40.38% | 42 | 52.88% | 55 | 104 |
| Histological Response | 6.45% | 6 | 21.51% | 20 | 72.04% | 67 | 93 |

| Supplementary Table 2: Critical and important outcomes for Ulcerative Colitis raw data  Number of participants who answered = 115 | | | | | | | |
| --- | --- | --- | --- | --- | --- | --- | --- |
|  | Critical | | Important | | Do not consider | | Total |
| Clinical Remission | 81.42% | 92 | 18.58% | 21 | 0.00% | 0 | 113 |
| Endoscopic Remission | 67.86% | 76 | 23.21% | 26 | 8.93% | 10 | 112 |
| Serious Adverse Events | 66.36% | 73 | 23.64% | 26 | 10.00% | 11 | 110 |
| Clinical Response | 51.38% | 56 | 34.86% | 38 | 13.76% | 15 | 109 |
| Withdrawal due to Adverse Events | 47.06% | 48 | 30.39% | 31 | 22.55% | 23 | 102 |
| Biochemical Remission | 32.04% | 33 | 38.83% | 40 | 29.13% | 30 | 103 |
| Histological Remission | 31.53% | 35 | 37.84% | 42 | 30.63% | 34 | 111 |
| Endoscopic Response | 27.36% | 29 | 49.06% | 52 | 23.58% | 25 | 106 |
| Total Adverse Events | 18.37% | 18 | 33.67% | 33 | 47.96% | 47 | 98 |
| Histological Response | 9.47% | 9 | 26.32% | 25 | 64.21% | 61 | 95 |
| Biochemical Response | 6.38% | 6 | 37.23% | 35 | 56.38% | 53 | 94 |
| Radiological Response | 3.30% | 3 | 12.09% | 11 | 84.62% | 77 | 91 |
| Radiological Remission | 2.15% | 2 | 17.20% | 16 | 80.65% | 75 | 93 |

| Supplementary Table 3: Ranking clinical scoring system for active Crohn's disease in order of preference | | | | | | | | |
| --- | --- | --- | --- | --- | --- | --- | --- | --- |
|  | 1 | | 2 | | 3 | | Total | Score |
| Harvey Bradshaw Index (HBI)  > 5 | 49.00% | 49 | 32.00% | 32 | 19.00% | 19 | 100 | 2.3 |
| Crohn's Disease Activity Index (CDAI) > 150 | 30.00% | 30 | 31.00% | 31 | 39.00% | 39 | 100 | 1.91 |
| Crohn's Disease Patient Reported Outcomes 2 (CD PRO2) | 21.00% | 21 | 37.00% | 37 | 42.00% | 42 | 100 | 1.79 |
|  | | | | | | | **Answered** | **100** |
|  |  |  |  |  |  |  | **Skipped** | **31** |

| Supplementary Table 4: Ranking biochemical scoring system for active Crohn's disease in order of preference | | | | | | |
| --- | --- | --- | --- | --- | --- | --- |
|  | 1 | | 2 | | Total | Score |
| CRP > 5 | 11.46% | 11 | 88.54% | 85 | 96 | 1.11 |
| Faecal Calprotectin > 250 | 88.54% | 85 | 11.46% | 11 | 96 | 1.89 |
|  | | | | | **Answered** | **96** |
|  |  |  |  |  | **Skipped** | **35** |

| Supplementary Table 5: Ranking endoscopic scoring system for active Crohn's disease in order of preference | | | | | | |
| --- | --- | --- | --- | --- | --- | --- |
|  | 1 | | 2 | | Total | Score |
| Simple Endoscopic Score for Crohn's Disease (SES CD) >3 | 73.21% | 41 | 26.79% | 15 | 56 | 1.73 |
| Crohn's Disease Index of Severity (CDEIS) > 3 | 26.79% | 15 | 73.21% | 41 | 56 | 1.27 |
|  | | | | | **Answered** | **56** |
|  |  |  |  |  | **Skipped** | **75** |

| Supplementary Table 6: Ranking clinical scoring system for active UC in order of preference | | | | | | | | | |
| --- | --- | --- | --- | --- | --- | --- | --- | --- | --- |
|  | 1 | | 2 | | 3 | Total | | | Score |
| modified Partial Mayo Score > 2 | 41.11% | 37 | 46.67% | 42 | 12.22% | | 11 | 90 | 2.29 |
| Simple Clinical Colitis Activity Index (SCCAI) | 42.22% | 38 | 20.00% | 18 | 37.78% | | 34 | 90 | 2.04 |
| Ulcerative Colitis Patient Reported Outcomes 2 (UC PRO2) | 16.67% | 15 | 33.33% | 30 | 50.00% | | 45 | 90 | 1.67 |
|  | | | | | | **Answered** | | | **90** |
|  |  |  |  |  |  | **Skipped** | | | **41** |

| Supplementary Table 7: Ranking biochemical scoring system for active UC in order of preference | | | | | | |
| --- | --- | --- | --- | --- | --- | --- |
|  | 1 | | 2 | | Total | Score |
| CRP > 5 | 5.26% | 5 | 94.74% | 90 | 95 | 1.05 |
| Faecal Calprotectin > 250 | 94.74% | 90 | 5.26% | 5 | 95 | 1.95 |
|  | | | | | **Answered** | **95** |
|  |  |  |  |  | **Skipped** | **36** |

| Supplementary Table 8: Ranking endoscopic scoring system for active UC in order of preference | | | | | | |
| --- | --- | --- | --- | --- | --- | --- |
|  | 1 | | 2 | Total | | Score |
| Mayo Score > 1 | 39.06% | 25 | 60.94% | 39 | 64 | 1.39 |
| Ulcerative Colitis Endoscopic Score Index of Severity (UCEIS) > 2 | 60.94% | 39 | 39.06% | 25 | 64 | 1.61 |
|  | | | | **Answered** | | **64** |
|  |  |  |  | **Skipped** | | **67** |

| Supplementary Table 9: Thresholds Means, Standard deviations (SD) and number of responses |
| --- |
| \|  \| Trivial to Small \| SD \| Number of Responses \| Small to Moderate \| SD \| Number of Responses \| Moderate to Large \| SD \| Number of Responses \| \| --- \| --- \| --- \| --- \| --- \| --- \| --- \| --- \| --- \| --- \| \| Clinical Remission \| 10.89 \| 6.02 \| 98 \| 19.96 \| 8.47 \| 98 \| 31.23 \| 13.23 \| 98 \| \| Clinical Response \| 13.88 \| 6.85 \| 96 \| 24.14 \| 9.78 \| 97 \| 36.08 \| 14.74 \| 97 \| \| Endoscopic Remission \| 9.44 \| 4.88 \| 87 \| 17.39 \| 9.3 \| 87 \| 27.94 \| 13.55 \| 87 \| \| Endoscopic Response \| 12.53 \| 6.63 \| 86 \| 21.69 \| 9.35 \| 86 \| 32.65 \| 13.5 \| 86 \| \| Biochemical Remission \| 12.84 \| 6.76 \| 85 \| 22.45 \| 10.73 \| 85 \| 34.58 \| 15.8 \| 85 \| \| Biochemical Response \| 14.61 \| 8.2 \| 85 \| 25.01 \| 11.71 \| 85 \| 37.62 \| 16.06 \| 85 \| \| Radiological Remission \| 10.53 \| 5.21 \| 81 \| 19.63 \| 8.89 \| 81 \| 30.35 \| 14.07 \| 81 \| \| Radiological Response \| 13.17 \| 6.64 \| 82 \| 23.34 \| 10.62 \| 82 \| 34.65 \| 14.78 \| 82 \| \| Histological Remission \| 9.96 \| 5.12 \| 83 \| 18.54 \| 9 \| 83 \| 28.82 \| 14.57 \| 83 \| \| Histological Response \| 12.35 \| 6.98 \| 82 \| 22.12 \| 11.05 \| 82 \| 32.94 \| 15.71 \| 82 \| \| Withdrawal to Adverse Events \| 7.09 \| 5.03 \| 90 \| 13.81 \| 8.42 \| 90 \| 22.6 \| 13.71 \| 90 \| \| Serious Adverse Events \| 5.91 \| 5.92 \| 90 \| 10.92 \| 8.71 \| 90 \| 16.98 \| 12.24 \| 90 \| \| Total Adverse Events \| 9.01 \| 6.08 \| 88 \| 16.19 \| 10.15 \| 88 \| 24.44 \| 14.28 \| 88 \| |

| Supplementary Table 10: **Responses from BSG (UK).** Thresholds Means, Standard deviations (SD) and number of responses | | | | | | | | | |
| --- | --- | --- | --- | --- | --- | --- | --- | --- | --- |
|  | Trivial to Small | SD | Number of Responses | Small to Moderate | SD | Number of Responses | Moderate to Large | SD | Number of Responses |
| Clinical Remission | 10.51 | 5.34 | 63 | 20.37 | 8.97 | 63 | 31.4 | 14.04 | 63 |
| Clinical Response | 13.35 | 6.83 | 62 | 24.05 | 9.9 | 62 | 35.74 | 14.41 | 62 |
| Endoscopic Remission | 8.69 | 4.77 | 54 | 17.3 | 10.5 | 54 | 27.7 | 14.46 | 54 |
| Endoscopic Response | 11.79 | 5.91 | 53 | 21.32 | 9.32 | 53 | 32.32 | 13.48 | 53 |
| Biochemical Remission | 11.5 | 5.77 | 52 | 21.13 | 10.65 | 52 | 33.21 | 16.48 | 52 |
| Biochemical Response | 13 | 6.47 | 52 | 23.31 | 10.53 | 52 | 35.6 | 15.64 | 52 |
| Radiological Remission | 9.8 | 4.75 | 50 | 18.98 | 8.82 | 50 | 29.98 | 14.82 | 50 |
| Radiological Response | 12.2 | 5.9 | 50 | 22.2 | 9.81 | 50 | 33.32 | 14.66 | 50 |
| Histological Remission | 9.37 | 4.84 | 52 | 17.85 | 9.02 | 52 | 28.1 | 15.33 | 52 |
| Histological Response | 11.83 | 6.68 | 52 | 21.6 | 10.75 | 52 | 32.4 | 15.49 | 52 |
| Withdrawal to Adverse Events | 6.89 | 5.09 | 57 | 13.68 | 9.2 | 57 | 22.63 | 14.78 | 57 |
| Serious Adverse Events | 5.88 | 6.8 | 57 | 10.84 | 9.84 | 57 | 16.93 | 13.71 | 57 |
| Total Adverse Events | 8.59 | 6.02 | 56 | 15.45 | 10.28 | 56 | 23.96 | 14.98 | 56 |

| Supplementary Table 11: **Responses from Europe**. Thresholds Means, Standard deviations (SD) and number of responses | | | | | | | | | |
| --- | --- | --- | --- | --- | --- | --- | --- | --- | --- |
|  | Trivial to Small | SD | Numbr of Responses | Small to Moderate | SD | Number of Responses | Moderate to Large | SD | Number of Responses |
| Clinical Remission | 12.81 | 5.58 | 16 | 20.25 | 5.99 | 16 | 31.56 | 10.57 | 16 |
| Clinical Response | 17.13 | 7.97 | 15 | 25.38 | 10.33 | 16 | 36.56 | 16.61 | 16 |
| Endoscopic Remission | 12.4 | 4.79 | 15 | 18.73 | 4.95 | 15 | 28.13 | 8.03 | 15 |
| Endoscopic Response | 16.33 | 8.44 | 15 | 23.27 | 9.45 | 15 | 32.27 | 12.01 | 15 |
| Biochemical Remission | 17.21 | 8.19 | 15 | 25.67 | 9.98 | 15 | 35.67 | 12.81 | 15 |
| Biochemical Response | 19.67 | 11.18 | 15 | 28.13 | 13.09 | 15 | 38.93 | 15.36 | 15 |
| Radiological Remission | 12.67 | 5.61 | 15 | 20.53 | 6.16 | 15 | 29.2 | 9.33 | 15 |
| Radiological Response | 17.2 | 7.64 | 15 | 25.4 | 10.21 | 15 | 34.67 | 13.6 | 15 |
| Histological Remission | 10.81 | 4.98 | 15 | 17.67 | 5.26 | 15 | 25.2 | 6.97 | 15 |
| Histological Response | 12.47 | 6.5 | 15 | 20.33 | 8.95 | 15 | 27.93 | 11.46 | 15 |
| Withdrawal to Adverse Events | 8.93 | 6.13 | 15 | 15.07 | 7.25 | 15 | 21.67 | 9.25 | 15 |
| Serious Adverse Events | 6 | 4.58 | 15 | 10.73 | 6.41 | 15 | 15.47 | 6.25 | 15 |
| Total Adverse Events | 10.33 | 5.66 | 15 | 18.2 | 8.08 | 15 | 24.87 | 11.2 | 15 |

| Supplementary Table 12: **Responses from North America**. Thresholds Means, Standard deviations (SD) and number of responses | | | | | | | | | |
| --- | --- | --- | --- | --- | --- | --- | --- | --- | --- |
|  | Trivial to Small | SD | Numbr of Responses | Small to Moderate | SD | Number of Responses | Moderate to Large | SD | Number of Responses |
| Clinical Remission | 10.53 | 7.89 | 19 | 18.37 | 8.37 | 19 | 30.42 | 12.44 | 19 |
| Clinical Response | 13 | 4.96 | 19 | 23.42 | 8.76 | 19 | 36.79 | 14.08 | 19 |
| Endoscopic Remission | 9.22 | 4.31 | 18 | 16.56 | 8.05 | 18 | 28.5 | 14.37 | 18 |
| Endoscopic Response | 11.56 | 5.76 | 18 | 21.44 | 9.21 | 18 | 33.94 | 14.62 | 18 |
| Biochemical Remission | 13.06 | 6.55 | 18 | 23.56 | 10.86 | 18 | 37.61 | 15.57 | 18 |
| Biochemical Response | 15.06 | 7.98 | 18 | 27.33 | 12.76 | 18 | 42.39 | 16.71 | 18 |
| Radiological Remission | 10.81 | 5.61 | 16 | 20.81 | 10.85 | 16 | 32.56 | 15.1 | 16 |
| Radiological Response | 12.47 | 6.44 | 17 | 24.88 | 12.62 | 17 | 38.53 | 15.44 | 17 |
| Histological Remission | 11.13 | 5.81 | 16 | 21.63 | 10.87 | 16 | 34.56 | 15.66 | 16 |
| Histological Response | 14.07 | 8.1 | 15 | 25.73 | 13.05 | 15 | 39.8 | 17.66 | 15 |
| Withdrawal to Adverse Events | 6.17 | 3.11 | 18 | 13.17 | 6.33 | 18 | 23.28 | 13.24 | 18 |
| Serious Adverse Events | 5.94 | 3.39 | 18 | 11.33 | 6.16 | 18 | 18.39 | 10.85 | 18 |
| Total Adverse Events | 9.24 | 6.44 | 17 | 16.88 | 11.01 | 17 | 25.65 | 14.31 | 17 |
